# Functional Characterization of the Venus Flytrap Domain of the Human TAS1R2 Sweet Taste Receptor

**DOI:** 10.3390/ijms23169216

**Published:** 2022-08-16

**Authors:** Anni Laffitte, Christine Belloir, Fabrice Neiers, Loïc Briand

**Affiliations:** Centre des Sciences du Goût et de l’Alimentation, CNRS, INRAE, Institut Agro, Université de Bourgogne Franche-Comté, 21000 Dijon, France

**Keywords:** G-protein-coupled receptor (GPCR), sugar, sweetener, sweet taste, taste, class C GPCR

## Abstract

The human sweet taste receptor is a heterodimeric receptor composed of two distinct G-protein-coupled receptors (GPCRs), TAS1R2 and TAS1R3. The TAS1R2 and TAS1R3 subunits are members of a small family of class C GPCRs whose members share the same architecture, comprising a Venus Flytrap (VFT) module linked to the seven transmembrane domains (TMDs) by a short cysteine-rich region (CRR). The VFT module of TAS1R2 contains the primary binding site for most of the sweet-tasting compounds, including natural sugars and artificial and natural sweeteners. However, cellular assays, molecular docking and site-directed mutagenesis studies have revealed that the VFT, CRR and TMD of TAS1R3 interact with some sweeteners, including the sweet-tasting protein brazzein. The aim of this study was to better understand the contribution of TAS1R2-VFT in the binding of sweet stimuli. To achieve this, we heterologously expressed human TAS1R2-VFT (hTAS1R2-VFT) in *Escherichia coli*. Circular dichroism spectroscopic studies revealed that hTAS1R2-VFT was properly folded with evidence of secondary structures. Using size-exclusion chromatography coupled with light scattering, we found that hTAS1R2-VFT behaves as a monomer. Ligand binding quantified by intrinsic tryptophan fluorescence showed that hTAS1R2-VFT is capable of binding sweet stimuli with K_d_ values, in agreement with physiological detection. Furthermore, we investigated whether the impact of point mutations, already shown to have deleterious effects on cellular assays, could impact the ability of hTAS1R2-VFT to bind sweet ligands. As expected, the ligand affinities of hTAS1R2-VFT were drastically reduced through the introduction of single amino acid substitutions (D278A and E382A) known to abolish the response of the full-length TAS1R2/TAS1R3 receptor. This study demonstrates the feasibility of producing milligram quantities of hTAS1R2-VFT to further characterize the mechanism of binding interaction and perform structural studies.

## 1. Introduction

Sweet-tasting compounds are detected by a heterodimeric receptor composed of two G protein-coupled receptors (GPCRs), named TAS1R2 (taste receptor type 1, member 2) and TAS1R3 (taste receptor type 1), expressed at the surface of the taste receptor cells in the taste buds [1,2,3]. These subunits are members of the small class C GPCR family. All members of this family share similar architectures made of a large extracellular domain composed of the Venus Flytrap (VFT) domain linked to the transmembrane domain (TMD) by a shorter rigid cysteine-rich region (CRR) [4,5,6]. The sweet taste receptor is activated by a wide repertoire of chemically distinct sweet compounds, including sugars, polyols, natural and artificial sweeteners and rare sweet-tasting plant proteins [4,7,8,9]. The presence of multiple binding sites on the TAS1R2/TAS1R3 taste receptor explains the synergy observed between some sweetener mixtures [10,11,12]. The human VFT of TAS1R2 (hTAS1R2-VFT) contains the primary binding site of sweet compounds, where sugars (sucrose, glucose and fructose) and noncaloric sweeteners (sucralose, aspartame, neotame, saccharin, acesulfame-K and steviosides) interact [1,13,14,15,16,17,18]. The human TAS1R3-VFT module (hTAS1R3-VFT) has been demonstrated to bind natural sugars (sucrose and glucose) and sucralose [16,19], showing that these compounds target TAS1R2 and TAS1R3 subunits, with different binding affinities. In addition, it has been found that hTAS1R3-TMD interacts with cyclamate and neohesperidin dihydrochalcone [20,21]. Finally, hTAS1R2-TMD has been shown to bind the natural and synthetic sweeteners perillartine and S-819 [12,22,23,24].

One of the major limitations in studying the molecular mechanisms of ligand binding is the difficulty of producing the whole sweet receptor complex in large quantities with high purity and conformational integrity to perform biophysical studies. To circumvent this problem, different strategies have been developed that allow us to understand the mechanisms of ligand detection and receptor activation. Thus, mouse and human TAS1R2- and TAS1R3-VFTs overexpressed using *Escherichia coli* have both been shown to interact with natural sugars and chlorodeoxysugar sucralose with distinct and physiologically relevant affinities [16,19]. It has also been demonstrated that hTAS1R2-VFT fused with the small ubiquitin-like modifier (SUMO) is expressed in bacteria as a functional protein. Combining various biophysical approaches and NMR, hTAS1R2-VFT was demonstrated to interact with the artificial sweetener neotame with a K_d_ value in the micromolar range [25]. More recently, the whole hTAS1R2 subunit overexpressed in a HEK293S inducible cell line was capable of binding high-potency sweeteners with K_d_ values that are in agreement with physiological detection [26].

We previously reported a strategy to express in bacteria pure and functional human TAS1R3-VFT able to bind sucralose with a binding affinity in the millimolar range [19]. This protocol was improved to study the ligand-binding properties of the VFT of the cat TAS1R1 (cTAS1R1-VFT) umami taste receptor [27]. Biophysical studies have demonstrated that cTAS1R1-VFT is capable of binding L-amino acids with K_d_ values in the micromolar range. In addition, we observed, as expected, that the 5′-monophosphate ribonucleotide IMP potentiated L-amino acid binding, further demonstrating that the protein was properly refolded and functional. To further understand the structural basis of sweet-tasting compound recognition by the sweet taste receptor, we extended this study to hTAS1R2-VFT. The target protein was expressed in inclusion bodies with a very high yield, as previously reported [28]. The protein was refolded, and the correct formation of secondary structures was verified using circular dichroism (CD). Using intrinsic tryptophan fluorescence, we demonstrated that hTAS1R2-VFT was able to interact with natural sugars and various noncaloric sweeteners with physiologically relevant affinity. To demonstrate that the binding is specific, we performed alanine substitutions (D278A and E382A) in the binding site of hTAS1R2-VFT. The methodology described here provides new tool for measuring binding affinity with sweeteners or enhancers and should help understanding the individual contribution of TAS1R2-VFT to receptor functionality.

## 2. Results

### 2.1. Expression and Purification of Human TAS1R2-VFT

**Figure 1:**
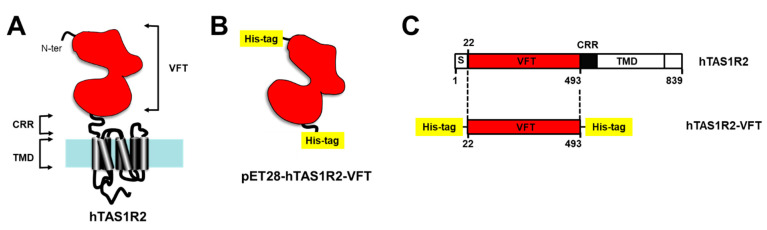
Strategy used for the expression of hTAS1R2-VFT in bacteria. (**A**) The Venus Fly Trap domain (VFT) of human TAS1R2 was expressed independently from the transmembrane domain (TMD), minus a short putative signal peptide (S) and a cysteine-rich region (CRR). (**B**) The pET28-hTAS1R2-VFT vector encodes a fusion protein that contains an N-terminal His-tag cleavable with thrombin, followed by hTAS1R2-VFT (Ala22-Ser493) and a C-terminal His-tag. (**C**) Full-length hTAS1R2 is represented according to its primary amino acid sequence deduced from the DNA sequence. The numerical positions of amino acid residues of hTAS1R2 are indicated.

**Figure 2:**
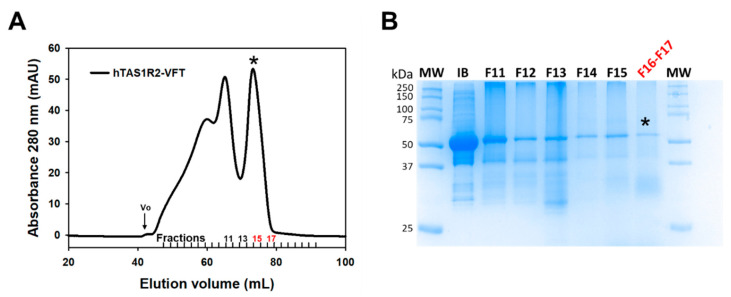
Size exclusion chromatography of purified hTAS1R2-VFT. (**A**) SEC analysis was performed on an Akta Pure FPLC system equipped with a Superdex 200 16/600 preparative grade column (GE Healthcare). Purified hTAS1R2-VFT was eluted using 50 mM Tris-HCl pH 8, 150 mM NaCl, 0.5 mM DDM, and 1 mM DTT at 1 mL/min. The last peak eluted at approximately 73.4 mL was analysed by (**B**) SDS–PAGE followed by staining with Coomassie blue. The fraction numbers refer to those designated in (**A**). Initial inclusion bodies (IB) used for refolding were loaded on SDS–PAGE as a control. Fractions F16 and F17, marked with a black asterisk, contained mainly hTAS1R2-VFT, used for subsequent measurements.

To investigate the structural basis of sweetener recognition by the human TAS1R2/TAS1R3 taste receptor, we expressed large quantities of recombinant hTAS1R2-VFT using the *E. coli* prokaryotic expression system as previously described [19,28] (Figure 1 and Figure S1). We found that hTAS1R2-VFT was only detected in the insoluble fraction as inclusion bodies (IBs) (Figure S2A). IBs were isolated and purified as described in the experimental section. SDS–PAGE analysis of purified hTAS1R2-VFT IBs revealed a major band migrating at a molecular mass of approximately 50 kDa (Figure 2B and Figure S2B), in agreement with the theoretical molecular mass values (57 kDa) calculated from the protein sequence. Approximately 200 mg of hTAS1R2-VFT IBs was obtained from 1 L of bacterial culture. hTAS1R2-VFT was then refolded using the protocol previously reported for cTAS1R1-VFT [27]. The isolation of the monomeric refolded protein was performed using preparative gel filtration chromatography. The chromatogram shows two distinct peaks: the first one eluted at 61.9 mL, whereas the second one eluted at approximately 73.4 mL (Figure 2A). SDS–PAGE (Figure 2B) and Western blot analysis (Figure S2B) revealed that purified hTAS1R2-VFT protein was mainly present in the peak eluted at approximately 73.4 mL, corresponding to a molecular weight of 73 kDa, as calculated from the gel filtration calibration curve (Figure S3). These data suggest that refolded hTAS1R2-VFT behaves as a monomeric form. The slight increase in molecular weight could be due to the presence of detergent molecules around the protein, which disrupts the electrophoretic migration. The corresponding protein peak fraction detected by Coomassie blue staining also revealed that hTAS1R2-VFT was more than 90% pure (Figure 2B). The expression and purification of hTAS1R2-VFT mutants (D278A and E382A) followed the same expression and purification protocol and led to the same SEC chromatogram as for wild-type protein (Figure S4). From 20 mg of purified IBs, we obtained approximately 1.2 mg of each refolded hTAS1R2-VFT.

### 2.2. Characterization of hTAS1R2-VFT

**Figure 3:**
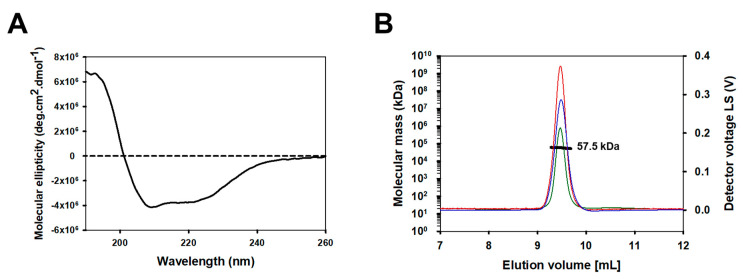
Secondary structure and oligomerization analysis of the purified hTAS1R2-VFT. Size exclusion chromatography of purified hTAS1R2-VFT. (**A**) The far-UV circular dichroism spectrum of hTAS1R2-VFT, recorded with 50 mM Tris-HCl pH 8, 150 mM NaCl, 0.5 mM DDM and 1 mM DTT, shows that there is a high content of α-helical secondary structures. Protein concentration—1 mg/mL. Light path—0.01 cm. (**B**) For SEC-MALS analysis, purified hTAS1R2-VFT was injected into a Superdex 200 10/300, 24 mL column (GE Healthcare) and eluted with 50 mM Tris-HCl pH 8, 150 mM NaCl, and 0.5 mM DDM. The chromatograms show the calculated molecular mass (bold black curve), differential refractive index dRI (blue curve), light scattering (red curve) and UV at 280 nm (green curve). hTAS1R2-VFT has a fitted molecular mass of 57.5 kDa; its theoretical monomer molecular mass value is 57.0 kDa.

To confirm the folding and structural integrity of refolded hTAS1R2-VFT, we used CD spectroscopy, a spectrophotometric method, to assess the secondary structure content of proteins in solution. The far-UV CD spectrum of hTAS1R2-VFT displayed a positive peak centred at 193 nm and two negative peaks at 208 and 222 nm, characteristic of a folded protein containing α-helical secondary structures (Figure 3A). CD spectrum deconvolution revealed that hTAS1R2-VFT is composed of approximately 72% α-helices and 9% β-sheets. These proportions were approximately the same for TAS1R2-VFT mutants (Table S1).

Online size-exclusion chromatography (SEC) coupled with multiangle light scattering (MALS), refractive index detector (RI) and UV measured at 280 nm allowed us to determine the oligomerization state of hTAS1R2-VFT purified in fractions 16 and 17 during preparative gel filtration. SEC-MALS analysis revealed the presence of hTAS1R2-VFT in a homogenous form, with an average molecular weight of 57.5 kDa (Figure 3B). The theoretical mass of hTAS1R2-VFT is 57.0 kDa; thus, the refolded hTAS1R2-VFT appeared mainly as a monomer. The linear and cumulative distribution of the molar mass confirmed that the monomer make up more than 98% of the total amount.

### 2.3. Ligand Binding Properties of hTAS1R2-VFT

**Figure 4:**
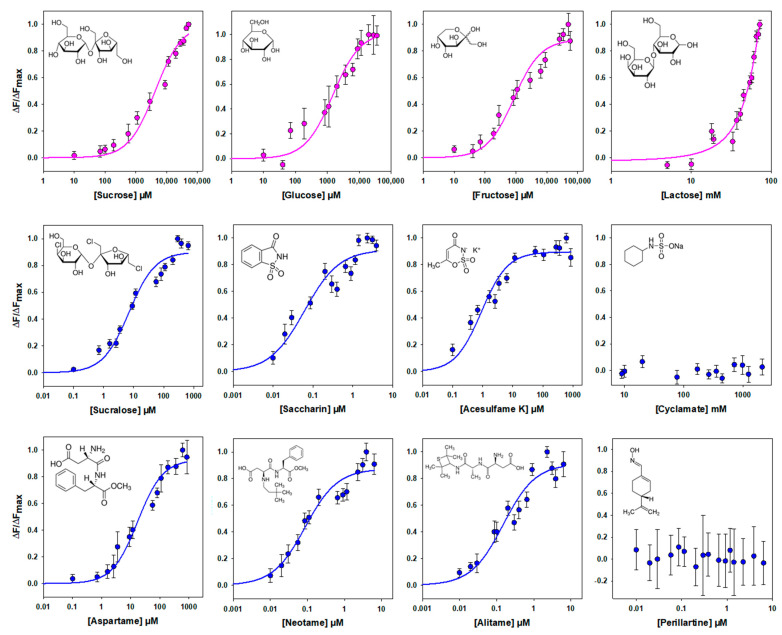
Binding properties of hTAS1R2-VFT assessed by intrinsic tryptophan fluorescence. Titration curves of hTAS1R2-VFT were generated with natural sugars (pink circles) or sweeteners (blue circles). Circles show experimental data, while the solid lines are the computed binding curves; excitation and emission wavelengths were 295 nm and 340 nm, respectively; hTAS1R2-VFT concentration, 0.5 µM. Data points correspond to the means ± SEMs of at least 12 independent replicates of at least three independently refolded protein samples, except for glucose, 6 independent replicates over three independently refolded protein samples.

Different sweeteners, including sugars and noncaloric sweeteners, have been shown to bind to the VFT of human TAS1R2 [1,13,14,15,16,17,18,25]. Therefore, we tested the binding affinity of hTAS1R2-VFT using an intrinsic tryptophan fluorescence assay. This approach allows one to measure protein–ligand interactions by measuring the fluorescence emitted by tryptophan residues upon the titration of sweet ligands. This technique is very sensitive to changes in the local environment of fluorescent amino acid residues within a protein and has been used to measure protein–ligand binding interactions with mouse, human and cat TAS1R-VFTs [16,19,27]. For this purpose, we determined the concentration response relationships for the intrinsic tryptophan fluorescence of hTAS1R2-VFT upon the addition of various sweet compounds. As shown in Figure 4, we found that natural sugars and artificial sweeteners induced conformational changes in hTAS1R2-VFT, leading to saturable intrinsic fluorescence enhancement. As previously observed with mouse TAS1R2-VFT [16], titration curves revealed that natural sugars were the ligands with the lowest affinity, exhibiting K_d_ values in the millimolar range. In contrast, we observed that artificial sweeteners bind with the highest affinities hTAS1R2-VFT, leading to K_d_ values in the nano-/micromolar range (Table 1). In the case of lactose, at high concentrations, the binding curve does not clearly form a plateau phase. This lack of a plateau may be attributed to the poor aqueous solubility of lactose at the highest concentrations and its low sweetness potency. The sweeteners cyclamate and perillartine were used as negative controls because cyclamate is known to bind hTAS1R3-TMD and perillartine interacts with the TMD of hTAS1R2 [20,22,24]. As expected, the addition of both sweeteners did not induce any fluorescence changes, showing that these compounds do not interact with hTAS1R2-VFT.

**Figure 5:**
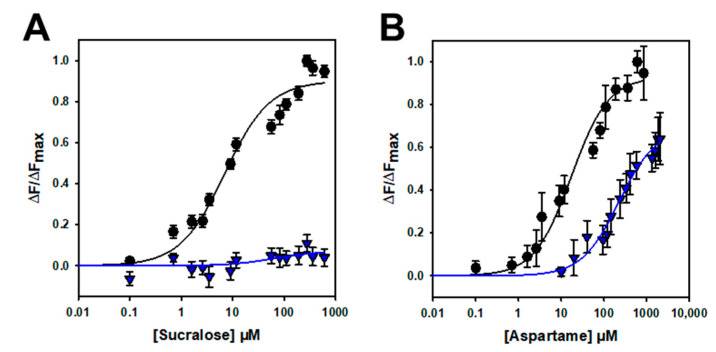
Titration curves of hTAS1R2-VFT-D278A (blue triangles) and hTAS1R2-VFT (black circles) with sucralose (**A**) and aspartame (**B**). Circles and triangles show experimental data, while the solid lines are the computed binding curves. Wild-type receptor dose–responses are the same as for Figure 4. Data points correspond to the means ± SEMs of at least 12 independent replicates of at least three independently refolded protein samples.

**Figure 6:**
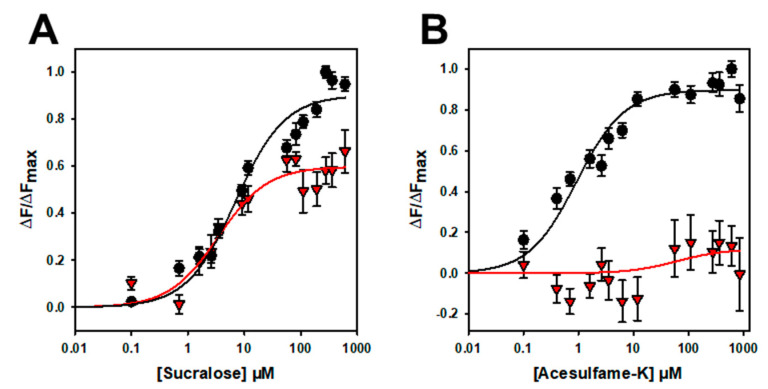
Titration curves of hTAS1R2-VFT-D382A (red triangles) and hTAS1R2-VFT (black circles) with sucralose (**A**) and acesulfame K (**B**). Circles and triangles show experimental data, while the solid lines are the computed binding curves. Wild-type receptor dose–responses are the same as for Figure 4. Data points correspond to the means ± SEMs of at least *n* = 12 independent replicates of at least three independently refolded protein samples.

To further confirm that the binding is specific, we performed mutagenesis studies on hTAS1R2-VFT. Using a cellular-based assay, the substitution of D278 to A (D278A) has been shown to abolish the response of the full-length hTAS1R2/hTAS1R3 receptor for sucralose and decrease the response for aspartame [15]. We performed ligand-binding titration assays in the same way as for hTAS1R2-VFT for the two point mutants. As expected, we found that hTAS1R2-VFT-D278A completely lost its affinity towards sucralose but was still able to bind aspartame, with a reduced affinity compared to hTAS1R2-VFT (Figure 5; Table 2). Cellular assays showed that the substitution of E382A reduces the full-length hTAS1R2/hTAS1R3 receptor response for sucralose and abolishes that for acesulfame-K [15]. We tested the ability of hTAS1R2-VFT-E382A to bind sucralose and acesulfame-K using an intrinsic tryptophan fluorescence assay. We observed that hTAS1R2-VFT-E382A binds sucralose with both a reduced affinity (K_d_ values) and amplitude, whereas the affinity to acesulfame-K was fully abolished (Figure 6; Table 2). Taken together, these data clearly demonstrate that hTAS1R2-VFT isolated from inclusion bodies is functional and that the binding is specific.

## 3. Discussion

The sweet taste receptor subunits (TAS1R2 and TAS1R3) are class C GPCRs [29]. These subunits assemble to form a unique receptor involved in the detection of chemically diverse sweet stimuli [1,2,3]. Sweet-tasting compounds include natural sugars, artificial and natural sweeteners, and rare plant sweet-tasting proteins [4]. Here, we describe the overexpression of the VFT domain of human TAS1R2 in bacteria, which was successfully obtained in large quantities as a soluble and functional protein. This study extends the characterization of the human TAS1R3-VFT protein that we previously described [19]. CD demonstrated that hTAS1R2-VFT was properly folded and contained secondary structures that are consistent with those measured on mouse and human TAS1R-VFTs [16,19]. In contrast with hTAS1R3-VFT, which was observed to be a dimer [19], gel filtration and SEC-MALS analysis revealed that hTAS1R2-VFT behaves as a monomer, as previously observed with cTAS1R1-VFT [27]. These data indicate that the VFT domain of hTAS1R2 is stable in the absence of hTAS1R3-VFT. This observation contrasts with TAS1R2-VFT expressed in the SUMO fusion protein, which was observed to exist in the dimeric state [25].

Our intrinsic fluorescence data demonstrate that hTAS1R2-VFT is functional and able to bind chemically diverse sweet stimuli. We determined its affinity constants for natural sugars using dose–response curves. We found that hTAS1R2-VFT interacts with the natural sugars sucrose, fructose and glucose, with K_d_ values in the millimole range, as previously observed with mouse TAS1R2-VFT [16]. Conversely, we observed that lactose is a poor ligand for hTAS1R2-VFT, in agreement with its low sweetness potency deduced from sensory experiments [30,31]. Our study revealed that hTAS1R2-VFT bind various noncaloric sweeteners with different affinities, in agreement with their sweetness potencies (Table 1). The binding is specific because hTAS1R2-VFT does not bind perillartine and cyclamate, which have been demonstrated to interact with TAS1R2- and TAS1R3-TMD, respectively [20,22,24]. Interestingly, the measured K_d_ values for all tested compounds were lower than the predicted values deduced from the half maximal effective concentration (EC_50_) values from the human TAS1R2/TAS1R3 receptor measured using a cellular-based assay or from sensory experiments conducted on human panels (Table 1). These K_d_ values are lower than those measured for the whole hTAS1R2 subunit [26]. This may be due to the impact of the presence of TMD. Nie et al. have already reported slightly lower K_d_ values for mouse TAS1R2-VFT compared to predicted values deduced from rodent behavioural studies or receptor functional assays [16]. Interestingly, our fluorescence-based assay revealed that hTAS1R2-VFT binds saccharin and acesulfame-K with K_d_ values much lower than would be expected from the EC_50_ value measured using a cellular-based assay (Table 1). One explanation is the presence of inhibitory binding sites for saccharin and acesulfame-K on the sweet taste receptor, which are probably located within the TAS1R3-TMD [15,32].

To further demonstrate that the binding is specific, we investigated the impact of two amino acid residue substitutions on the binding properties of hTAS1R2-VFT. Cellular-based assays have shown that the two substitutions D278A and E382A differentially affect the response of the full-length TAS1R2/TAS1R3 receptor for sucralose, aspartame or acesulfame-K [15]. Our ligand binding titration assay demonstrated that hTAS1R2-VFT-D278A completely lost its affinity towards sucralose, whereas the affinity of hTAS1R2-VFT-E382A for acesulfame-K was fully abolished (Table 2).

In conclusion, our data clearly demonstrate that hTAS1R2-VFT refolded from inclusion bodies is functional and able to specifically bind some sweet compounds at physiologically relevant concentrations. Our data on hTAS1R2-VFT point mutants confirmed that the loss of functional activity measured in the cellular assay does occur at the receptor level and is not the consequence of a modulation of the signalling pathway. This study provides new insights into the molecular determinants of sweet taste perception and opens the way to screen new sweet-tasting compounds or taste modulators.

## 4. Materials and Methods

### 4.1. Chemicals

Ligands were obtained from commercial sources as follows: sucrose, fructose, glucose, sucralose, aspartame, saccharin, acesulfame-K, cyclamate, neotame and alitame were obtained from Sigma–Aldrich (Saint-Quentin Fallavier, France) with a purity greater than or equal to 98% and preserved following the manufacturer’s instructions. The solutions for the dose–response analysis were prepared in 50 mM Tris-HCl solution at pH 8.0 and kept frozen at −20 °C until use. *N*-dodecyl-β-D-maltopyranoside (DDM) was purchased from Anatrace (Affymetrix, High Wycombe, UK). Mouse monoclonal anti-His antibody and goat anti-mouse horseradish peroxidase conjugated secondary antibody were purchased from BioRad (Marnes-la-Coquette, France).

### 4.2. Design of TAS1R2 Expression Constructs

The cDNA sequence encoding the *Homo sapiens* TAS1R2-VFT minus a putative signal sequence and the CRR was synthesized by DNA 2.0 (Newark, CA, USA), optimized for expression in *E. coli*, and subcloned into the pET28a vector (Novagen, Darmstadt, Germany). The resulting expression plasmid pET28-hTAS1R2-VFT encodes a fusion protein comprising an N-terminal His_6_-tag that can be cleaved by thrombin, followed by hTAS1R2-VFT (Ala22-Ser493) and another His_6_-tag at the C-terminus (Figure S1). The plasmid was subsequently transformed into *E. coli* DH5 alpha cells, and colonies were selected on LB agar plates containing kanamycin. The plasmid DNA from selected colonies was purified using a Macherey-Nagel kit (NucleoSpin Plasmid Mini Kit from Macherey Nagel, Hoerdt, France) and used as a template for further site-directed mutagenesis.

hTAS1R2-VFT point mutants were constructed using PCR-based mutagenesis (QuikChange Multi Site-Directed Mutagenesis kit, Agilent Technologies, Les Ulis, France) using the following primers: forward primer 5′-GTTGTTGTTTTTAGTCCGGCTCTGACCCTGTATCACTTT-3′; reverse primer 5′-AAAGTGATACAGGGTCAGAGCCGGACTAAAAACAACAAC-3′ for hTAS1R2-VFT D278A and 5′-AGTTTGGATTGCAAGCGCAAGCTGGGCAATTGATC-3′; 5′-GATCAATTGCCCAGCTTGCGCTTGCAATCCAAACT-3′ for hTAS1R2-VFT E382A. PCR conditions were as follows: 1 min at 95 °C; 15 cycles of 0.5 min at 59 °C, 2 min at 72 °C and 1 min at 95 °C; followed by 10 min at 72 °C. Each mutated plasmid was transformed into *E. coli* DH5alpha cells, amplified and purified. The integrity of all constructs was verified by automated DNA sequencing (Genewiz).

### 4.3. Protein Expression and Inclusion Bodies Production

The chemically competent *E. coli* One Shot^®^ BL21 Star™ (DE3) (Invitrogen, Life Technologies, Illkirch-Graffenstaden, France) was transformed with the pET28-hTAS1R2-VFT vector, and mutants were constructed. The method used for the production of inclusion bodies (IBs) by IPTG induction and the following steps of washing, refolding, purification and biophysical characterization were performed as previously described for cat TAS1R1 with slight modifications [27].

Briefly, IB expression was induced with a 1 mM final concentration of isopropyl β-D-1-thiogalactopyranoside (IPTG) for 20 h at 20 °C in 2YT medium supplemented with 45 µg/mL kanamycin. Induced cells were harvested by centrifugation, resuspended in lysis buffer (50 mM Tris-HCl pH 8.0, 10 mM EDTA, 100 mM NaCl, 0.5% (*v*/*v*) Triton X-100, 0.2% Tween 20, 5% glycerol, 1 mM PMSF, DNase I), disrupted by sonication, and centrifuged. The pellet containing IB was washed two times in the presence of 4 M urea and once more resuspended in 50 mM Tris-HCl pH 8, 150 mM NaCl, 0.5% Triton X-100 and 10 mM EDTA, then dispatched into small aliquots in 1 mL microtubes and centrifuged at 24,000× *g* at 4 °C for 30 min. The supernatant was discarded, and the pellets were carefully dried before flash freezing with liquid nitrogen. The washed and dry IBs were conserved at −20 °C until use.

### 4.4. In Vitro Refolding of TAS1R2-VFTs

In the same way, hTAS1R2-VFT was refolded by dialysis using a protocol previously improved for cTAS1R1-VFT [27] with slight modifications. Briefly, one sample of 20 mg of IBs was resuspended in 1 mL of solubilizing buffer (50 mM Tris-HCl pH 8.0, 20 mM SDS, 8 M urea, 25 mM DTT), mixed with borosilicate glass beads of 3 mm diameter and incubated at 50 °C for 2 h at 600 rpm. The solubilized IBs were then centrifuged at 25,000× *g* for 15 min at 15 °C. The IBs were then diluted 200 times in a dilution buffer (50 mM Tris-HCl pH 8, 20 mM SDS, 8 M urea, 12.5 mM DTT) and further solubilized in a water bath at 50 °C for 1 h to allow complete unfolding of the protein. The IBs were then dialyzed twice (1:8 *v*/*v* ratio) in a Spectrapor dialysis membrane (Spectrum Laboratories, New Brunswick, NJ, USA) with a 10 kDa cut-off against a first buffer (50 mM Tris-HCl pH 8, 20 mM SDS, 1 M urea, 1 mM DTT) for 1 h 30 min and then overnight against a second buffer (50 mM Tris-HCl pH 8, 2 mM SDS, 25 mM DTT). After dialysis, the protein was concentrated 8 times using a concentrator (Vivaspin) with a 50 kDa cut-off (Startorius, Göttingen, Germany), and the obtained suspension was filtered with a 0.2 µm filter. First, a 5 mM final concentration of DDM was carefully added to the dialyzed and concentrated suspension. The refolding was obtained with a fast precipitation of SDS with the addition of 300 mM NaCl and 300 mM KCl under vigorous magnetic agitation. The suspension was then centrifuged at 48,400× *g* for 30 min at 15 °C, and the supernatant obtained was filtered at 0.2 µm. The remaining filtrate was dialyzed overnight at a 1:45 ratio against the following buffer (50 mM Tris-HCl pH 8, 150 mM NaCl, 150 mM KCl, 0.5 mM DDM, 1 mM DTT).

Refolded hTAS1R2-VFT was concentrated to 2 mg/mL using concentrators with a 10 kDa cut-off (Vivaspin). To separate the monomeric receptor from aggregates, gel filtration was performed using a 120 mL preparative grade Superdex 200 16/600 column (GE Healthcare, Chicago, IL, USA) connected to a Äkta Pure FPLC system (GE Healthcare, Chicago, IL, USA). The column was equilibrated with 50 mM Tris-HCl pH 8, 150 mM NaCl, 0.5 mM DDM, and 1 mM DTT at 1 mL/min. One or two injections of 5 mL of hTAS1R2-VFT were performed.

Sample aliquots taken during purification were analysed by SDS–PAGE (10% Mini-PROTEAN^®^ TGX™ precast polyacrylamide gels, BioRad, Marnes-la-Coquette, France). using Coomassie staining and Western blot analysis. For Western blot analysis, samples were separated by SDS–PAGE and then transferred to PolyVinyliDene Fluoride (PVDF) membranes (BioRad) and immunoblotted with primary monoclonal mouse anti-His antibody (MCA1396, Bio-Rad) diluted to 1/10,000 followed by goat anti-mouse horseradish peroxidase-conjugated antibody (#31430, Life Technologies, 1/50,000). Detection was performed using ECL chemiluminescent substrate (Clarity ECL Western Blotting kit, BioRad) and a ChemiDoc imaging system (Bio-Rad). Eluted fractions containing monomeric protein revealed by SDS–PAGE analysis were pooled and measured for absorbance at 280 nm to calculate the protein concentration.

### 4.5. Circular Dichroism

CD spectra were recorded using a JASCO J-815 spectropolarimeter (Jasco, Tokyo, Japan) equipped with a Peltier temperature controller. The TAS1R2-VFTs obtained after gel filtration were concentrated to 1 mg/mL in 50 mM Tris-HCl pH 8, 150 mM NaCl, 0.5 mM DDM, and 1 mM DTT. The spectra were recorded at 20 °C using a 0.01 cm path length quartz cell between 190 and 260 nm at 0.5 nm intervals with a 50 nm/min scan speed. Data were averaged over 10 accumulated scans. The buffer contributions were subtracted, and the data were converted to mean residue ellipticity in deg·cm^2^·dmol^−1^. Secondary structure proportions were computed using the deconvolution CDSSTR algorithm on DICHROWEB (accessed on 1 June 2017, http://dichroweb.cryst.bbk.ac.uk/html/home.shtml).

### 4.6. SEC-MALS Analysis

The oligomeric state of the purified hTAS1R2-VFT protein was measured using a size exclusion chromatography (SEC) column (Superdex 200 10/300 from GE Healthcare, Velizy-Villacoublay, France) coupled to a triple-angle light scattering detector (Mini- DAWN™ TREOS, Wyatt Technology), a differential refractometer (RID-10A, Shimadzu, Kyoto, Japan) and a SpectraSeries UV100 detector (Thermo Separation Products, Waltham, MA, USA). The molecular weight was determined using ASTRA VI software, version 6.1 (Wyatt Technology, Santa Barbara, CA, USA) with a dn/dc value of 0.185 mL/g.

### 4.7. Intrinsic Tryptophan Fluorescence Analysis

All measurements were conducted at 20 °C using a Cary Eclipse spectrofluorimeter (Agilent Technologies, Santa Clara, CA, USA) equipped with a Peltier temperature control unit with a 5 nm bandwidth for emission and excitation. Ligand binding experiments with 0.5 µM hTAS1R2-VFTs were performed in buffer composed of 50 mM Tris-HCl pH 8, 150 mM NaCl, 0.5 mM DDM and 1 mM DTT. The intrinsic fluorescence of hTAS1R2-VFTs was measured at an excitation wavelength of 295 nm, and emission spectra were recorded from 320 to 380 nm. Fluorescence emission values were collected at 340 nm wavelength in the presence and absence of sweet ligands. A range of ligand concentrations were freshly prepared in 50 mM Tris-HCl pH 8.0. For titration experiments, successive 0.4 µL aliquots of sweeteners were added to 400 µL of hTAS1R2-VFT solution. Fluorescence measurements were corrected for bleaching, dilution and nonspecific buffer quenching. The dissociation constants (K_d_) were calculated from a plot of the ratio between the fluorescence intensity variation and the maximum fluorescence intensity variation (ΔF/ΔFmax) versus the concentration of the total ligand. Assuming that the binding of hTAS1R2-VFT to sweet compounds is described by a one-site binding model, the K_d_ values of ligand–receptor interactions were determined with a standard nonlinear regression method using SigmaPlot 12.5 software (Systat Software, Inc., San Jose, CA, USA) as previously described [27]. For each ligand, the experiment was repeated on at least twelve cells over at least three different batches of refolded hTAS1R2-VFT.

## Figures and Tables

**Table 1:** K_d_ values of hTAS1R2-VFT measured by intrinsic fluorescence for each sweetener compared to the EC_50_ value obtained from cellular assays and estimated sweetness potency.

Sweetener	hTAS1R2-VFT (K_d_)	EC_50_	Sweetness Potency *
Lactose	>70 mM	n.d.	0.3 ^f^
Glucose	1.5 ± 0.3 mM	n.d.	0.6 ^f^
Fructose	0.9 ± 0.2 mM	8 ± 4 mM ^b^	0.7 ^e^
Sucrose	4.2 ± 0.9 mM	11 ± 3 mM ^b^	1 ^e^
Cyclamate	n.b.	767 ± 83 μM ^c^	26 ^e^
Acesulfame-K	0.9 ± 0.2 μM	213 ± 59 μM ^c^	200 ^e^
Aspartame	17.2 ± 3.1 μM	75 ± 11 μM ^d^	250 ^e^
Saccharin	0.06 ± 0.02 μM	190 ± 7 μM ^d^	500 ^e^
Sucralose	7.0 ± 1.3 μM	36 ± 2 μM ^b^	600 ^e^
Perillartine	n.b.	2.54 ± 0.48 µM ^c^	1000 ^g^
Alitame	0.15 ± 0.03 μM	13.8 ± 0.3 μM ^a^	4500 ^e^
Neotame	0.09 ± 0.02 μM	0.90 ± 0.09 μM ^c^	11,000 ^g^

* Sweetness potency is described on a molar basis. ^a^ Unpublished data obtained from collaborators); ^b^ unpublished data from our laboratory or from ^c^ Belloir et al., 2021; ^d^ Masuda et al., 2012; ^e^ Schiffman and Gatlin, 1993; in water equivalent to 2% sucrose; ^f^ Shallenberger, 1993; in water equivalent to 5% sucrose; ^g^ Bassoli et al., 2002; in water equivalent to 2% sucrose. n.b., no binding. n.d., not determined.

**Table 2:** Apparent K_d_ values measured by intrinsic tryptophan fluorescence for hTAS1R2-VFT and mutants D278A and E382A.

Sweetener	hTAS1R2-VFT	hTAS1R2-VFT-D278A	hTAS1R2-VFT-E382A
Sucralose	7.0 ± 1.3 μM	n.b. *	3.5 ± 0.8 μM
Acesulfame-K	0.9 ± 0.2 μM	n.d. *	n.b.
Aspartame	17.2 ± 3.1 μM	222.8 ± 27.7 μM	n.d.

* n.b. no binding; n.d. not determined.

## Data Availability

All the data in this paper are expressed as the means, and the error bars indicate the standard deviations. For the visualization of the results, we used Sigmaplot software. The datasets generated and analysed during the present study are available from the corresponding author on request.

## Supplementary Materials

The following supporting information can be downloaded at https://www.mdpi.com/article/10.3390/ijms23169216/s1.

## References

[1] Li X., Staszewski L., Xu H., Durick K., Zoller M., Adler E. (2002). Human receptors for sweet and umami taste. Proc. Natl. Acad. Sci. USA.

[2] Nelson G., Chandrashekar J., Hoon M.A., Feng L., Zhao G., Ryba N.J., Zuker C.S. (2002). An amino-acid taste receptor. Nature.

[3] Nelson G., Hoon M.A., Chandrashekar J., Zhang Y., Ryba N.J., Zuker C.S. (2001). Mammalian sweet taste receptors. Cell.

[4] Belloir C., Neiers F., Briand L. (2017). Sweeteners and sweetness enhancers. Curr. Opin. Clin. Nutr. Metab. Care.

[5] DuBois G.E. (2016). Molecular mechanism of sweetness sensation. Physiol. Behav..

[6] Servant G., Kenakin T., Zhang L., Williams M., Servant N. (2020). The function and allosteric control of the human sweet taste receptor. Adv. Pharmacol..

[7] Behrens M. (2022). Pharmacology of TAS1R2/TAS1R3 Receptors and Sweet Taste. Handb. Exp. Pharmacol..

[8] Behrens M., Meyerhof W. (2018). Vertebrate Bitter Taste Receptors: Keys for Survival in Changing Environments. J. Agric. Food Chem..

[9] Neiers F., Belloir C., Poirier N., Naumer C., Krohn M., Briand L. (2021). Comparison of Different Signal Peptides for the Efficient Secretion of the Sweet-Tasting Plant Protein Brazzein in Pichia pastoris. Life.

[10] Fujiwara S., Imada T., Nakagita T., Okada S., Nammoku T., Abe K., Misaka T. (2012). Sweeteners interacting with the transmembrane domain of the human sweet-taste receptor induce sweet-taste synergisms in binary mixtures. Food Chem..

[11] Jang J., Kim S.K., Guthrie B., Goddard W.A. (2021). Synergic Effects in the Activation of the Sweet Receptor GPCR Heterodimer for Various Sweeteners Predicted Using Molecular Metadynamics Simulations. J. Agric. Food Chem..

[12] Servant G., Tachdjian C., Li X., Karanewsky D.S. (2011). The sweet taste of true synergy: Positive allosteric modulation of the human sweet taste receptor. Trends Pharmacol. Sci..

[13] Assadi-Porter F.M., Maillet E.L., Radek J.T., Quijada J., Markley J.L., Max M. (2010). Key amino acid residues involved in multi-point binding interactions between brazzein, a sweet protein, and the T1R2-T1R3 human sweet receptor. J. Mol. Biol..

[14] Maillet E.L., Cui M., Jiang P., Mezei M., Hecht E., Quijada J., Margolskee R.F., Osman R., Max M. (2015). Characterization of the Binding Site of Aspartame in the Human Sweet Taste Receptor. Chem. Senses.

[15] Masuda K., Koizumi A., Nakajima K., Tanaka T., Abe K., Misaka T., Ishiguro M. (2012). Characterization of the modes of binding between human sweet taste receptor and low-molecular-weight sweet compounds. PLoS ONE.

[16] Nie Y., Vigues S., Hobbs J.R., Conn G.L., Munger S.D. (2005). Distinct contributions of T1R2 and T1R3 taste receptor subunits to the detection of sweet stimuli. Curr. Biol. CB.

[17] Xu H., Staszewski L., Tang H., Adler E., Zoller M., Li X. (2004). Different functional roles of T1R subunits in the heteromeric taste receptors. Proc. Natl. Acad. Sci. USA.

[18] Zhang F., Klebansky B., Fine R.M., Liu H., Xu H., Servant G., Zoller M., Tachdjian C., Li X. (2010). Molecular mechanism of the sweet taste enhancers. Proc. Natl. Acad. Sci. USA.

[19] Maitrepierre E., Sigoillot M., Le Pessot L., Briand L. (2012). Recombinant expression, in vitro refolding, and biophysical characterization of the N-terminal domain of T1R3 taste receptor. Protein Expr. Purif..

[20] Jiang P., Cui M., Zhao B., Snyder L.A., Benard L.M., Osman R., Max M., Margolskee R.F. (2005). Identification of the cyclamate interaction site within the transmembrane domain of the human sweet taste receptor subunit T1R3. J. Biol. Chem..

[21] Winnig M., Bufe B., Kratochwil N.A., Slack J.P., Meyerhof W. (2007). The binding site for neohesperidin dihydrochalcone at the human sweet taste receptor. BMC Struct. Biol..

[22] Cai C., Jiang H., Li L., Liu T., Song X., Liu B. (2016). Characterization of the Sweet Taste Receptor Tas1r2 from an Old World Monkey Species Rhesus Monkey and Species-Dependent Activation of the Monomeric Receptor by an Intense Sweetener Perillartine. PLoS ONE.

[23] Servant G., Tachdjian C., Tang X.Q., Werner S., Zhang F., Li X., Kamdar P., Petrovic G., Ditschun T., Java A. (2010). Positive allosteric modulators of the human sweet taste receptor enhance sweet taste. Proc. Natl. Acad. Sci. USA.

[24] Zhao M., Xu X.-Q., Meng X.-Y., Liu B. (2018). The Heptahelical Domain of the Sweet Taste Receptor T1R2 Is a New Allosteric Binding Site for the Sweet Taste Modulator Amiloride That Modulates Sweet Taste in a Species-Dependent Manner. J. Mol. Neurosci..

[25] Assadi-Porter F.M., Radek J., Rao H., Tonelli M. (2018). Multimodal Ligand Binding Studies of Human and Mouse G-Coupled Taste Receptors to Correlate Their Species-Specific Sweetness Tasting Properties. Molecules.

[26] Belloir C., Brulé M., Tornier L., Neiers F., Briand L. (2021). Biophysical and functional characterization of the human TAS1R2 sweet taste receptor overexpressed in a HEK293S inducible cell line. Sci. Rep..

[27] Belloir C., Savistchenko J., Neiers F., Taylor A.J., McGrane S., Briand L. (2017). Biophysical and functional characterization of the N-terminal domain of the cat T1R1 umami taste receptor expressed in Escherichia coli. PLoS ONE.

[28] Maitrepierre E., Sigoillot M., Le Pessot L., Briand L. (2013). An efficient Escherichia coli expression system for the production of a functional N-terminal domain of the T1R3 taste receptor. Bioengineered.

[29] Behrens M., Briand L., de March C.A., Matsunami H., Yamashita A., Meyerhof W., Weyand S. (2018). Structure-Function Relationships of Olfactory and Taste Receptors. Chem. Senses.

[30] Harrison S.K., Bernhard R.A. (1984). Time-Intensity Sensory Characteristics of Saccharin, Xylitol and Galactose and Their Effect on the Sweetness of Lactose. J. Food Sci..

[31] Schiffman S.S., Gatlin C.A. (1993). Sweeteners: State of knowledge review. Neurosci. Biobehav. Rev..

[32] Galindo-Cuspinera V., Winnig M., Bufe B., Meyerhof W., Breslin P.A. (2006). A TAS1R receptor-based explanation of sweet ‘water-taste’. Nature.

