# Supplementary material for: Functional Characterization of the Venus Flytrap Domain of the Human TAS1R2 Sweet Taste Receptor
Source: Int J Mol Sci. 2022 Aug 16;23(16):9216. doi: 10.3390/ijms23169216 (PMC9409066; doi:10.3390/ijms23169216)
Supplement: Supplementary file 1 [file ijms-23-09216-s001.zip › ijms-1813053-supplementary.pdf]

Functional characterization of the Venus Flytrap domain of the human TAS1R2 sweet taste receptor

Anni Laffitte, Christine Belloir, Fabrice Neiers and Loïc Briand *

Supplementary

```
        10         20         30         40         50         60
MGSSHHHHHH SSGLVPRGSH MAENSDFYLP GDYLLGGLFS LHANMKGIVH LNFLQVPMCK

        70         80         90        100        110        120
EYEVKVIGYN LMQAMRFAVE EINNDSSLLP GVLLGYEIVD VCYISNNVQP VLYFLAHEDN

       130        140        150        160        170        180
LLPIQEDYSN YISRVVAVIG PDNSESVMTV ANFLSLFLLP QITYSAISDE LRDKVRFPAL

       190        200        210        220        230        240
LRTTPSADHH IEAMVQLMLH FRWNWIIVLV SSDTYGRDNG QLLGERVARR DICIAFQETL

       250        260        270        280        290        300
PTLQPNQNMT SEERQRLVTI VDKLQQSTAR VVVVFSPDLT LYHFFNEVLR QNFTGAVWIA

       310        320        330        340        350        360
SESWAIDPVL HNLTELRHLG TFLGITIQSV PIPGFSEFRE WGPQAGPPPL SRTSQSYTCN

       370        380        390        400        410        420
QECDNCLNAT LSFNTILRLS GERVVYSVYS AVYAVAHALH SLLGCDKSTC TKRVVYPWQL

       430        440        450        460        470        480
LEEIWKVNFT LLDHQIFFDP QGDVALHLEI VQWQWDRSQN PFQSVASYYP LQRQLKNIQD

       490
ISWHTINNTI PMSHHHHHH
```

Figure S1. Amino acid sequence of hTAS1R2-VFT. The hTAS1R2-VFT sequence (Ala22-Ser493) is shown with a green background. Numbers refer to amino acid residues of hTAS1R2 (signal peptide: 1–19). His-Tags and thrombin cleavage sites are shown with yellow and pink backgrounds, respectively.

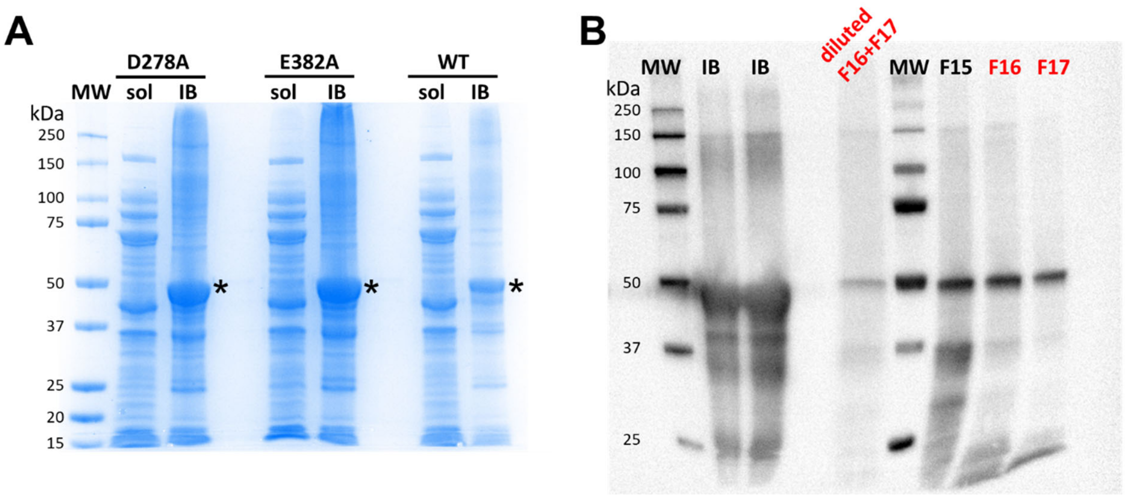

Figure S2. SDS–PAGE and western blot analysis of hTAS1R2-VFT wild-type (WT) and mutant hTAS1R2-VFT-D278A and hTAS1R2-VFT-D382A expressed in *E. coli* using the pET28 vector. (**A**) The proteins from the soluble fraction (sol) or insoluble fraction, known as inclusion bodies (IB), were separated by 10% acrylamide gels and stained with Coomassie blue. MW: molecular weight markers. Position of hTAS1R2-VFT is indicated by an asterisk. (**B**) Western blot analysis using mouse anti-His primary antibody and goat anti-mouse horseradish peroxidase conjugated secondary antibody revealed the presence of hTAS1R2-VFT wild-type in inclusion bodies (IB) (Lanes 1 and 2) and in the hTAS1R2-VFT wild-type purified fraction eluted by size exclusion chromatography from Figure 2.

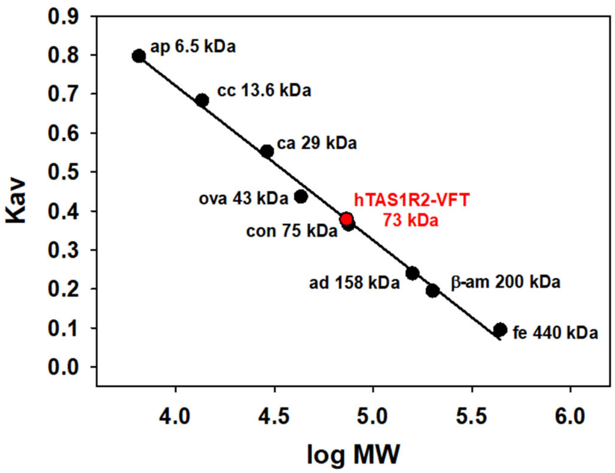

Figure S3. Gel filtration chromatography of purified hTAS1R2-VFT. The calibration curve for the HiLoad 16/600 Superdex 200 preparative grade column (GE Healthcare) was established with ferritin (fe, 440 kDa), β-amylase (β-am, 200 kDa), alcohol dehydrogenase (ad, 158 kDa), conalbumin (con, 75 kDa), ovalbumin (ova, 43 kDa), carbonic anhydrase (ca, 29 kDa), cytochrome c (cc, 13.6 kDa) and aprotinin (ap, 6.5 kDa). The estimated molecular mass of hTAS1R2-VFT is 73 kDa.

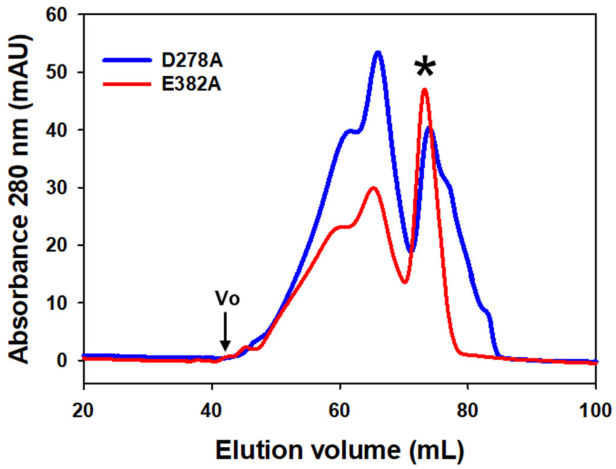

Figure S4. Preparative gel filtration chromatography of refolded hTAS1R2-VFT-D278A (solid blue line) and hTAS1R2-VFT-E382A (solid red line). The arrow indicates the position of the void volume (Vo), and the asterisk indicates the peak containing purified hTAS1R2-VFT. Gel filtration was performed using HiLoad 16/600 Superdex 200 preparative grade equilibrated with 50 mM Tris-HCl pH 8, 150 mM NaCl, 0.5 mM DDM, and 1 mM DTT at 1 mL/min.

Table S1. Secondary structure evaluation of the refolded hTAS1R2-VFT and mutants D278A and E382A.

Protein	α-helices (%)	ß-sheets (%)
hTAS1R2-VFT	72	9
hTAS1R2-VFT-D278A	66	13
hTAS1R2-VFT-E382A	69	13
